# Ultra-endurance athletic performance suggests that energetics drive human morphological thermal adaptation

**DOI:** 10.1017/ehs.2019.13

**Published:** 2019-12-13

**Authors:** Daniel P. Longman, Alison Macintosh Murray, Rebecca Roberts, Saskia Oakley, Jonathan C.K. Wells, Jay T. Stock

**Affiliations:** 1 School of Sport, Health and Exercise Sciences, Loughborough University, Loughborough LE11 3TU, UK; 2 Department of Anthropology, University of Victoria, British Columbia, Canada; 3 Department of Archaeology, University of Cambridge, Cambridge CB2 3QG, UK; 4 Childhood Nutrition Research Centre, UCL Institute of Child Health, London WC1N 1EH, UK; 5 Department of Anthropology, University of Western Ontario, Ontario, Canada; 6 Department of Archaeology, Max Planck Institute for the Science of Human History, Kahlaische Strasse 10, D-07745 Jena, Germany

**Keywords:** energetics, adaptation, thermoregulation, morphology, Bergmann's Rule, Allen's Rule

## Abstract

Both extinct and extant hominin populations display morphological features consistent with Bergmann's and Allen's Rules. However, the functional implications of the morphologies described by these ecological laws are poorly understood. We examined this through the lens of endurance running. Previous research concerning endurance running has focused on locomotor energetic economy. We considered a less-studied dimension of functionality, thermoregulation. The performance of male ultra-marathon runners (*n* = 88) competing in hot and cold environments was analysed with reference to expected thermoregulatory energy costs and the optimal morphologies predicted by Bergmann's and Allen's Rules. Ecogeographical patterning supporting both principles was observed in thermally challenging environments. Finishers of hot-condition events had significantly longer legs than finishers of cold-condition events. Furthermore, hot-condition finishers had significantly longer legs than those failing to complete hot-condition events. A degree of niche-picking was evident; athletes may have tailored their event entry choices in accordance with their previous race experiences. We propose that the interaction between prolonged physical exertion and hot or cold climates may induce powerful selective pressures driving morphological adaptation. The resulting phenotypes reduce thermoregulatory energetic expenditure, allowing diversion of energy to other functional outcomes such as faster running.

## Graphical abstract

**Figure:**
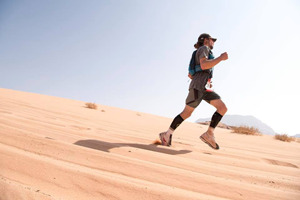

## Introduction

### Ecogeographical rules

Effective thermoregulation is a key challenge facing individuals in different environments. The apparent influence of climate on morphological traits has long been documented, and is an area of active research interest. Bergmann's and Allen's rules describe patterns of variation both within and across species in relation to the temperature of their environment. The two ecogeographic rules contend that endotherms will tend to be larger (Bergmann 1847) and display shorter limbs and body appendages (Allen 1887) in colder environments. Both rules are underpinned by the fundamental thermodynamic principles by which the body produces heat through cellular activity, such that heat generation is proportional to body mass, while heat loss through radiation, convection and evaporation is proportional to body surface area. As surface area increases as a square function of the linear dimension, and mass as a cubic function, an increase in body size increases the mass-to-surface area ratio. Large body size is thereby associated with a large heat-producing mass relative to the heat-losing surface area, and is favoured in colder conditions; in warmer conditions the inverse holds.

Both extinct and extant hominin species have been shown to display morphological features consistent with Bergmann's and Allen's rules (Foster and Collard 2013; Holliday 1997a, b; Holliday and Trinkaus 1991; Tilkens *et al*. 2007). Considering humans, early work by Roberts reported a negative association between annual temperature and body mass (Roberts 1953) and a positive association with leg length (Roberts 1973, 1978).

A plethora of research has provided broadly consistent support for these findings (Crognier 1981; Hiernaux 1968; Hiernaux and Fromont 1976; Ruff 1994; Stinson 1990; Trinkaus 1981). More recent analyses of previously published data concluded that humans conform to this pattern, although the trends at higher latitudes are getting weaker with time after 1950 (Katzmarzyk and Leonard 1998), and the pattern only holds when the range of temperature (or latitude) is sufficiently large (Foster and Collard 2013). In addition to absolute size, body composition also varies with temperature, with both higher heat-producing lean mass and high levels of energy-storing peripheral fat being associated with cold conditions in both sexes (Wells 2012). It has not yet been shown, however, if the morphologies predicted by Bergmann's and Allen's rules confer functional benefits in hot or cold environments. We tested this hypothesis by examining performance in ultra-endurance marathons in different climactic conditions.

### Physical activity, thermoregulation and morphological adaptation

Meaningful interactions have been demonstrated between physical activity and thermoregulation. The conversion of chemical energy to kinetic energy during muscular contraction is inefficient, with a substantial quantity of heat being produced (American College of Sports Medicine position stand 2007; Hawley *et al*. 2014). Through an assessment of energy allocation within physically active people in hot, temperate and cold climates, Ocobock (2016) demonstrated that the heat produced from exercise is sufficient to differentially influence thermoregulatory costs in hot and cold environments. In cold conditions, the heat produced by contracting muscles reduces the thermoregulatory burden and associated energetic costs. This supported previous laboratory-based work illustrating a decreased reduction in core body temperature when an individual immersed in cold water is exercising (Tikuisis *et al*. 2000). The opposite is true in hot conditions, where exercise-induced heat production increases thermoregulatory load and the risk of hyperthermia (Raynaud *et al*. 1976; Rivera-Brown *et al*. 2006).

Physical activity, in particular endurance running, may have played an important role in human evolution. Since its formulation in 1984, the endurance running hypothesis (Carrier 1984) has gained popularity and public recognition. The theory posits that the evolution of certain human traits can be explained as adaptations to selective pressures imposed by long-distance running. Research interest in this area stems from observations that, while humans perform poorly relative to other mammals and primates in terms of power, strength and sprinting speed, we are excellent endurance athletes. In contrast to other primate species, which are incapable of endurance running, humans are able to run distances of several kilometres using aerobic metabolism (Carrier 1984). Amateur human runners are able to sustain speeds of 5 m/s (Lieberman *et al*. 2006), which compares favourably with specialised quadrupedal cursors: a dog with a similar mass to a human (65 kg) has a trot–gallop transition speed of 3.8 m/s, and can then only sustain a gallop for a maximum of 15 min under ideal conditions (Heglund and Taylor 1988). Despite selective breeding for running ability, the same is true of horses (Minetti 2003). Consequently, the physical capacity for endurance running may have been a selected trait in our genus.

Selective pressures for running ability in early *Homo* may stem from the fitness benefits of quickly reaching scavenging sites or for hunting (Bramble and Lieberman 2004). While competition from other carnivores and a relative lack of scavenging opportunities suggest that scavenged meat could not have been depended upon by early hominins (Bunn 2001), the ability to run long distances and employ the technique of persistence hunting may have improved the chances of acquiring this rare, but valuable nutrient source (Lieberman *et al*. 2006). Recent evidence suggests that hunting ability (via its association with endurance running prowess) might serve as a reliable signal of mate fitness, in addition to its role in calorie provisioning (Longman *et al*. 2015). This may have led to a further selective pressure for running ability, and consequent morphological adaptations.

Research considering the functional implications of morphologies resultant from endurance running has predominantly focussed on locomotor energetic economy. In addition to skeletal features conferring increased balance and mechanisms for force stress dissipation while running (discussed in Bramble and Lieberman 2004), lower limb elongation in the *Homo* lineage (Will *et al*. 2017) is believed to have promoted locomotor economy. Longer legs are associated with greater economy in both the walking (Bramble and Lieberman 2004; Steudel-Numbers and Tilkens 2004) and running gaits (Pontzer 2007; Steudel-Numbers *et al*. 2007). Longer legs increase ground contact time (Roberts *et al*. 1998), decreasing the rate of ground force application and reducing the energetic cost of running (discussed in Wright and Weyand 2001). The main energetic cost of long legs, an increased limb mass moment of inertia, may be offset by the low stride frequency and compact feet of humans, which are smaller than those of chimpanzees when scaled to body mass (Zihlman and Brunker 1979).

Our study considers a new energetic dimension of leg functionality in endurance running – thermoregulation. The thermoregulatory demands placed upon the body by endurance activity are so strong that the largest physiological challenge that must be overcome by endurance runners is that of effective thermoregulation (El Helou *et al*. 2012; Lieberman and Bramble 2007). As the amount of heat generated during exercise is a function of the number and rate of muscular actin–myosin cross-bridges utilised for contraction, heat generation is significantly greater during running than walking, and increases with speed.

The predominant mechanism of heat loss in mammals is panting, which employs rapid shallow breaths at a rate far greater than normal breathing. However, when running (or galloping), the tight 1:1 coupling of striding and breathing prevents panting (Bramble and Jenkins 1993), thereby inhibiting cooling. Most mammals are thus forced to stop galloping shortly after beginning because panting cannot dissipate heat quickly enough to avoid hyperthermia. In contrast, humans do not have to couple breathing with running (Bramble and Carrier 1983) and an increased number and density of eccrine sweat glands facilitates rapid dissipation of heat by evaporation. Even in comparison with our closest relatives, the eccrine sweat density of humans is approximately 10-fold higher than in chimpanzees and macaques (Kamberov *et al*. 2018). These differences, in tandem with reduced body hair promoting convective heat loss and an upright gait reducing water stress in hot environments, allow human endurance running in hot conditions (Lieberman and Bramble 2007; Wheeler 1992). Movement may also play an important thermoregulatory role. By considering inter-segment differences in surface area, skin temperature and rate of movement, Cross and colleagues found that limb swing amplifies the cooling effect of evaporation (Cross *et al*. 2008). In this way, very subtle differences in limb dimensions could have a profound impact on cooling in the moving body.

Short-term biological adaptations to reduce the negative effects of heat stress are possible through the process of heat acclimatisation (Horowitz 2011; Sawka *et al*. 1996, 2011). Heat acclimatisation improves submaximal and maximal aerobic performance in hot conditions, and well has enhancing thermal comfort (Gonzalez and Gagge 1976; Lorenzo *et al*. 2010; Nielsen *et al*. 1993; Racinais *et al*. 2015). This is achieved via improvements to fluid-electrolyte balance, cardiovascular stability, skin blood flow response and sweating and reduced metabolic rate (Sawka *et al*. 2011). Around 7–14 days of heat exposure is required to acclimatise to the heat, with hot-condition exercise increasing the effectiveness. The benefits are short lived: cessation of heat exposure results in a loss of approximately 75% by the third week (Pandolf 1998). Athletes with higher levels of aerobic fitness are able to gain these benefits quicker, and retain them for longer (Armstrong and Pandolf 1988; Pandolf 1998).

Notwithstanding these adaptations, heat stress is a prominent challenge in human endurance running. Different optimal temperatures for peak performance in track and field events highlight the thermal challenge of dissipating excess heat during running. While the fastest sprinting and middle-distance (100–1500m) times are set at temperatures around 23°C (Haïda *et al*. 2013), the optimal ambient temperatures for the much longer marathon event are much lower, at 10–12°C (Maughan 2010). Such is the effect of temperature that a comprehensive analysis recently reported a quantifiable decrease in running speed with every 1°C increase from an optimal 9.9°C (El Helou *et al*. 2012). These findings no doubt informed Nike's and Ineos' decision to hold their recent attempts to break the 2 h barrier for the men's marathon in locations with temperatures in this range (Hutchinson 2017).

## The current study

The current study sought to utilise multi-day ultra-marathons in hot and cold environments to investigate whether the morphologies predicted by Bergmann's and Allen's rules confer a functional benefit in thermally challenging environments. This investigation stems from a wider research theme using contemporary sports as a tool to examine evolutionary theory (Longman *et al*. 2015, 2017a, 2018). More specifically, the current study is part of the ADaPt Project, which is developing the use of ultra-endurance challenges as experimental scenarios to study trade-offs relating to life history theory (Longman *et al*. 2017b).

Multi-day endurance events impose large energetic demands on both locomotion and thermoregulation. When combined with a reduced opportunity to consume adequate calories, the result is an energy deficit (Knechtle *et al*. 2005; Knechtle and Bircher 2005). The high metabolic demands of ultra-marathon competitions thereby provide an opportunity to investigate energetic trade-offs between competing and energetically demanding physiological functions (Longman *et al*. 2017b). Here, the functional implications for athletic performance of Bergmann's and Allen's long-standing ecogeographical rules will be considered.

Even in the absence of prolonged physical activity, thermoregulation imposes a significant energetic burden (Hill *et al*. 2013). In the cold, shivering can cause metabolic rate to be elevated to 5–6 times that observed at rest (Glickman *et al*. 1967; Iampietro *et al*. 1960; Keatinge *et al*. 1986). As morphological traits such as shorter leg length have been shown to reduce the metabolic cost of thermoregulation in the cold and ultimately resting metabolic rate (Tilkens *et al*. 2007), athletes exhibiting morphological traits which reduce the energetic burden of thermoregulation are expected to be capable of a greater energetic allocation towards running. In this way, climate-appropriate morphologies area expected to be associated with enhanced running performance. In the heat, an athlete's capacity to dissipate heat to the environment may restrict the maximal amount of energy they can expend in running (Speakman 2010). Those athletes with heat-adapted morphologies, which allow for increased rates of heat loss, are therefore expected to perform to a higher level. In both extremes of climate, the performance of athletes with morphologies suited to their environment are expected to benefit from increased energetic allocation towards running.

It is hypothesised that:

1. Athletes who successfully completed ultra-marathons in hot conditions will exhibit heat-adapted morphologies relative to those who successfully completed ultra-marathons in cold conditions.
2. Athletes who successfully completed a hot-condition ultra-marathon will exhibit a greater degree of ecogeographical heat-adaptation relative to athletes who failed to complete the same race.

**Figure 1.**
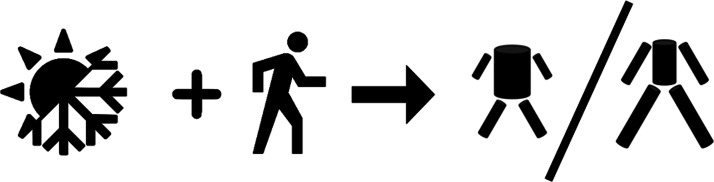
Proposed model: prolonged physical activity in thermally challenging environments provides the selective pressure for the generation of morphologies through natural selection or developmental plasticity.

If athletes with environmentally adapted morphologies were found to out-perform those without, the considerable thermoregulatory challenge of performing physical activity in hot and cold environments would be highlighted. Such a relationship might suggest that endurance activity in thermally challenging environments provides a selective pressure for the emergence of temperature-adapted morphologies; see Figure 1.

## Methods

Male athletes (*N* = 88) were recruited from the 2016 Rovaniemi150 (Finland), the 2016 Jungle Ultra (Peru), the 2016 and 2017 Al Andalus Ultimate Trail (Spain) and the 2016 Everest Trail Race (Nepal). These particular races were selected on the basis of their diverse range of climates and broad similarities in terms of race distance. Two races were considered to be hot (Peru and Spain), and the other two were considered to be cold (Finland and Nepal). With the exception of the Finland event, which was continuous, the remaining three events follow a similar format of five or six stages, run on consecutive days with overnight rest. An overview of the races can be seen below in Table 1.

**Table 1.** Overview of the four ultra-marathons

Race	Rovaniemi150,^a^ Finland	Jungle Ultra, Peru	Al Andalus Ultimate Trail, Spain	Everest Trail Race, Nepal
Location	Rovaniemi, Finland	Manu National Park, Peru	Granada Province, Spain	Solukhumbu District, Nepal
Latitude	66° N	12° S	37° N	27° N
Race dates	Late February	Early June	Mid-July	Mid-November
Race format	Continuous	5 stages	5 stages	6 stages
Distance	66/150 km	250 km	230 km	153 km
Elevation change	1356 m ascent, 1350 m descent	3200 m descent	7101 m ascent 7094 m descent	15,230 m ascent 14,280 m descent
Climate (daily range)	Cold (−11–2°C)	Hot and humid (26–34°C, 77%)	Hot and dry (35–45°C)	Cool days (5–15°C) Cold nights (−10°C)

^a^ Athletes were able to complete the Rovaniemi150 on foot, on skis or by bike. Only runners were included in this analysis.

One-way ANCOVAs were performed comparing all race entrants (irrespective of whether or not they finished) in hot-condition races with those in cold-condition races. While there were no significant differences in either weight or body mass index (BMI, body mass divided by the square of the height), significant differences were observed with leg length and relative leg length [leg length hot, 89.7 ± 4.2cm vs cold, 85.4 ± 4.8 cm, *F*(1,97) = 24.374, *p* < 0.001; relative leg length hot, 49.9 ± 1.3% vs cold, 48.0 ± 1.2%, *F*(1,97) = 55.779, *p* < 0.001). This suggests that there may have been a degree of self-selection (perhaps based on perceptions of differential ability in the hot and cold) with respect to Allen's, but not Bergmann's, Rule.

Athletes received an email explaining the study prior to race day, and were invited to participate. Measurements were taken 1–4 days prior to the race. Ethical approval for the project was granted by the University of Cambridge Human Biology Ethics Committee (HBREC.2016.14), and written informed consent was obtained.

### Anthropometrics

Bergmann's Rule has previously been assessed using a variety of measures. These include body mass, height, BMI and the ponderal index (body mass divided by the cube of the height) as well as hip and waist circumferences (Foster and Collard 2013; Ruff 1994). Each of these measures was taken in this study to examine the relationship between body size and race condition. Stature was measured to the nearest 0.1 cm using a Leicester Stadiometer. Waist circumference was taken at the level of the narrowest point between the lower costal (10th rib) border and the iliac crest. In cases where there was no obvious narrowing, the measurement was taken at the mid-point between the lower costal (10th rib) border and the iliac crest. Hip circumference was taken at the level of the greatest posterior protuberance of the buttocks, which usually corresponds anteriorly to about the level of the symphysis pubis. Sitting height was measured according to standards in International Standards of Anthropometric Assessment (2001). An index of leg length was obtained by subtracting sitting height from stature, and relative leg length was obtained by dividing leg length by stature.

### Defining athletes’ performance

Athletes’ performance within each race was categorised as finisher or non-finisher. Finishers were defined at those who completed the full race distance within the time limits set by race organisers, whereas non-finishers did not. The time limits are considered by athletes and organisers to be consistent across the races. This method of determining performance provided groups large enough for statistical analyses, and provided ecological validity owing to the high importance placed by athletes on reaching the set cut-off times.

### Statistics

To determine whether significant differences existed between race conditions, data collected from all participants and then from race finishers were analysed using a one-way analysis of covariance (ANCOVA), controlling for age. For graphical analysis, differences between groups were calculated in percentage terms, by multiplying the group-difference in natural log-transformed values of each trait by 100%.

To address the second hypothesis, one-way analysis of covariance (ANCOVA), controlling for age, was performed to compare finishing and non-finishing athletes within the Spanish race. All analyses were performed using SPSS v25, and significance set at *p* < 0.05.

The data is available online via https://www.lboro.ac.uk/departments/ssehs/staff/danny-longman/

## Results

A sample of 88 male athletes were tested across the two climatic conditions (hot and cold). The vast majority of the athletes were of European ethnic origin, and travelled to the country hosting the event. A description of the sample and race completion times, split by competition and condition, can be seen in Tables 2 and 3.

**Table 2.** Descriptive characteristics for all participating athletes, split by competition

	Finland mean (SD), *n* = 16	Peru mean (SD), *n* = 15	Spain mean (SD), *n* = 38	Nepal mean (SD), *n* = 19
Age (years)	38.1 (9.57)	41.5 (7.93)	49.2 (9.73)	46.3 (11.7)
Height (cm)	180.8 (5.09)	177.9 (4.45)	180.3 (6.85)	176.1 (5.96)
Weight (kg)	80.8 (5.45)	80.8 (7.38)	78.6 (8.76)	78.1 (10.0)
BMI (kg/m^2^)	24.7 (1.52)	25.5 (1.92)	24.1 (2.06)	25.2 (2.76)
Ponderal index	13.7 (1.00)	14.3 (1.13)	13.4 (1.30)	14.3 (1.70)
Waist circumference (cm)	80.9 (7.15)	85.4 (6.25)	85.6 (5.76)	85.7 (8.28)
Hip circumference (cm)	98.4 (5.13)	101.3 (3.39)	100.4 (5.33)	100.8 (5.42)
Leg length (cm)	86.12 (4.10)	88.6 (3.58)	90.2 (4.32)	84.9 (4.46)
Relative leg Length (%)	47.6 (1.18)	49.8 (1.16)	50.0 (1.40)	48.2 (1.12)

**Table 3.** Mean and standard deviations of the race time of finishers in hot- and cold-condition races

	Hot condition, mean (SD) (s)	Cold condition, mean (SD) (s)	*t*	*p*
	123,808.7 (19,208.3)	123,397.1 (82,320.5)	0.028	0.978

Independent samples *t*-tests were performed to assess whether acclimatisation and the morphological variables were linked. Acclimatisation to heat requires 7–14 days of heat exposure (Pandolf 1998), so athletes who arrived at the race location 7+ days prior to the race start were categorised as heat-acclimatised. Only two athletes were categorised as heat-acclimatised, and there were no significant differences in the morphologies between the heat-adapted and non-heat-adapted cohorts.

**Hypothesis 1**

Athletes who successfully completed ultra-marathons in hot conditions will exhibit heat-adapted morphologies relative to those who successfully completed ultra-marathons in cold conditions.

There is a body of literature investigating potentially important age effects on endurance running performance (Cejka *et al*. 2015; Hunter *et al*. 2011; Rüst *et al*. 2013, 2014). Indeed, race finishers were significantly younger than those who did not finish [42.9 ± 9.94 years vs 49.4 ± 10.5 years, *t*(86) = −2.922, *p* = 0.004]. Consequently, age was controlled for in analyses.

**Figure 2.**
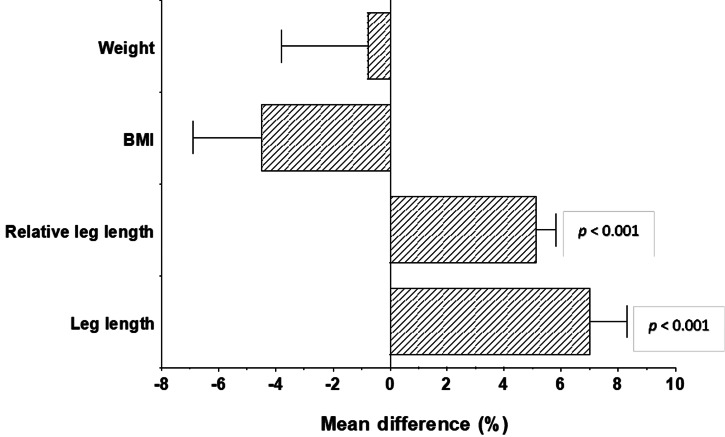
Chart showing percentage differences in anthropometric traits between finishers in hot and cold conditions. Positive values represent the variable being greater in hot-condition finishers than cold-condition finishers. The differences in relative leg length and leg length are statistically significant.

First, a one-way ANCOVA was conducted to determine statistically significant differences between the morphologies of finishers in hot (Spain and Peru) vs cold (Finland and Nepal) conditions, controlling for age. Athletes who finished a hot-condition race had longer leg lengths and greater relative leg length than athletes who finished a cold-condition race [leg length hot 90.6 ± 4.03cm vs cold 84.6 ± 4.02, *F*(3,82) = 9.862, *p* < 0.001; relative leg length hot 50.3 ± 0.977% vs cold 47.8 ± 1.03%, *F*(3,82) = 20.474, *p* < 0.001]. These results persisted following Bonferroni corrections (both *p* < 0.001), and were consistent with Allen's Rule. These results are summarised in Figure 2.

To further investigate the aforementioned possibility of self-selection to races, standardised variables for leg length and relative leg length were created by subtracting the mean of all entrants from the individual's measurement. One-way ANCOVAs were then performed to determine whether statistically significant differences existed between standardised measures (leg length and relative leg length) in hot and cold conditions. There were no significant differences between finishers in hot- and cold-conditions in any of these analyses (standardised leg length hot 0.885 ± 4.03 vs cold −0.885 ± 4.02, *p* = 0.621; standardised relative leg length hot 0.00375 ± 0.0098 vs cold −0.00195 ± 0.0103, *p* = 0.643). This lack of significant differences leads to the suggestion that self-selection may have played a significant role in an athlete's choice of competition.

**Hypothesis 2**

High-performing athletes within a hot race condition (Spain) will exhibit a greater degree of ecogeographical heat-adaptation relative to low-performing athletes in the same race.

Two consecutive editions of the Spain ultra-endurance event were investigated to obtain a sample size sufficient for this analysis. The Spain race was chosen for this further analysis because of the large sample size this event afforded, and because of the reduced cost of data collection in comparison to attending the other events. Race finishers were significantly younger than those who did not finish (45.0 ± 9.1 years vs 56.2 ± 5.8 years, *t*(36) = −4.228, *p* < 0.001). To address the second hypothesis, a one-way ANCOVA was conducted to determine statistically significant differences between the morphologies of finishers and non-finishers, controlling for age.

Consistent with differences between finishing athletes across conditions, athletes continued to show tentative trends in line with Allen's Rule, although not quite reaching significance. Athletes who finished the Spain race tended to, on average, exhibit larger leg length ratios (50.4 ± 1.0%) than those who did not [49.4 ± 1.8%, *F*(1,35) = 3.852, *p* = 0.058].

**Figure 3.**
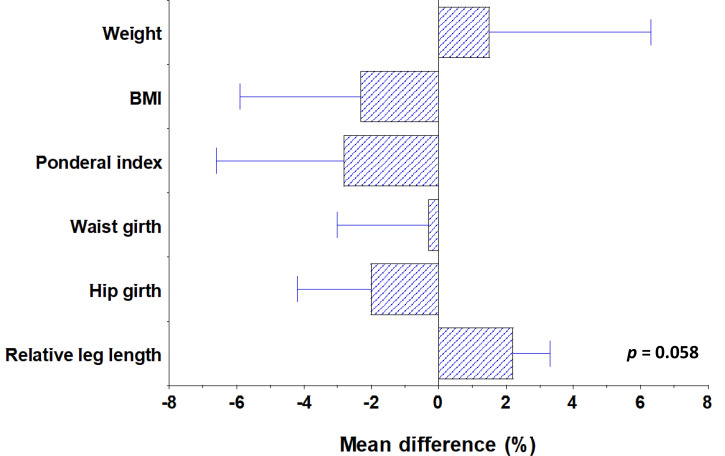
Chart showing percentage differences in anthropometric traits between finishers and non-finishers in a hot setting. Positive values represent the variable being greater in finishers than in non-finishers. The difference in relative leg length is borderline significant.

These results are visualised in Figure 3 and Table 4.

**Table 4.** Differences between finishers and non-finishers in the Spain ultra-marathon

	Finishers mean (SD), *n* = 24	Non-finishers mean (SD), *n* = 14	Comparison		
			d.f.	*F*	*p*
Height (cm)	180.9 (6.91)	179.3 (6.87)	1,35	0.074	0.788
Weight (kg)	78.9 (9.73)	78.0 (7.09)	1,35	0.108	0.744
BMI (kg/m^2^)	24.0 (1.90)	24.3 (2.37)	1,35	0.341	0.563
Ponderal index	13.3 (1.04)	13.6 (1.68)	1,35	0.363	0.551
Waist circumference	84.9 (5.00)	86.9 (6.89)	1,35	0.030	0.864
Hip circumference	100.0 (5.91)	101.0 (4.29)	1,35	0.798	0.378
Leg length (cm)	91.1 (4.06)	88.5 (4.42)	1,35	0.838	0.366
Relative leg length (%)	50.4 (.963)	49.4 (1.80)	1,35	3.852	0.058

## Discussion

It was hypothesised that ecogeographical patterning in human morphology would be observed in ultra-marathon runners successfully completing races in hot and cold environments. Heat-adapted morphologies are considered to be characterised by smaller bodies (Bergmann's Rule; as measured by mass, BMI, surface area: volume ratio or ponderal index; Foster and Collard 2013) and longer limbs (Allen's Rule). Such adaptations allow for a larger heat-dissipating surface area relative to heat-generating tissue mass, reducing thermal strain in a hot environment. In contrast, larger bodies and shorter limbs promote heat retention, and were hypothesised to be beneficial in the cold. As active thermoregulation is energetically costly (Hill *et al*. 2013), climate-appropriate morphology was expected to reduce this metabolic burden, allowing increased energetic allocation to running and enhanced performance in the cold. In the heat, increased capacity was expected to increase energetic alloction to running, and allow for faster running before inducing hyperthermia, enhancing running performance (Speakman 2010).

First, the characteristics of athletes entering hot vs cold-condition races were compared. Runners entering hot-condition races had greater leg lengths and greater relative leg lengths than runners entering cold-condition races. Other morphological variables did not differ. These differences may represent self-selection, in which a runner applies prior knowledge of their ability to tolerate hot or cold conditions to avoid exposing his or herself to environments in which they would expect to perform poorly. Indeed, the races chosen for this study are known within the ultra-running community to be particularly gruelling; they are advertised as such by race organisers. This apparent self-selection is consistent with Scarr and McCartney's (1983) niche-picking, in which individuals actively choose compatible environments which offer the best chance of success. Athletes entering these competitions may have perceived previous performance as appropriate to the climactic conditions of the races, without necessarily considering the mechanisms behind their previous experience.

Further support for the hypothesis and for the functional relevance of ecogeographical patterning came from analyses revealing that hot-condition finishers had longer leg lengths and longer relative leg lengths than cold-race finishers.

To test the second hypothesis we compared finishers with non-finishers in hot conditions. Athletes who finished the Spanish ultra-marathon had longer relative leg lengths than those who did not, although there were no significant differences in measures relating to Bergmann's Rule. The results of this within-race analysis suggest that, beyond any degree of self-selection discussed above, there existed a significant interactive effect of environment and morphology on performance once the race began. To perform well, adaptive morphologies appear to be required.

### Energetic implications of ecogeographical patterning

As discussed earlier, the relationship between limb morphology and locomotor energetic efficiencies is well documented. Lower limb elongation through the *Homo* lineage (Will *et al*. 2017) is believed to have promoted economy in both walking (Bramble and Lieberman 2004; Steudel-Numbers and Tilkens 2004) and running (Pontzer 2007; Steudel-Numbers *et al*. 2007). In parallel, there is a substantial body of literature dating back to the nineteenth century (Allen 1887) considering the thermoregulatory benefits of particular limb morphologies in different environments. However, to date the link between limb morphology and the energetics of both thermoregulation and locomotion has received relatively little attention.

Higgins and Ruff (2011) applied both thermoregulatory and locomotor energetic principles to consider Neanderthal lower limb morphology. Through trigonometric modelling the authors demonstrated that the truncated limb morphology of Neanderthals, historically considered to be a heat-conserving thermoregulatory adaptation to cold climates (Holliday and Ruff 2001; Roberts 1978; Ruff 1994; Trinkaus 1981), did not necessarily come at a cost to locomotor economy as previously thought (Steudel-Numbers and Tilkens 2004). This is due to Neanderthals’ shorter relative tibia length, allowing for an increased stride length on an uphill slope for a given hip excursion angle.

The present study further develops the idea of an energetic relationship between limb morphology, thermoregulation and locomotion. The differential effect of environmental temperature on the relationship between limb length and locomotor performance highlights the dynamic interplay between morphology and both energetically demanding processes. Previously, environmentally adapted morphologies have been shown to confer thermoregulatory energetic savings (Tilkens *et al*. 2007). It therefore seems reasonable to contend that athletes with morphologies aligned with the race environment benefitted from thermoregulatory energy savings. Energy that would otherwise have been consumed by thermoregulation was then available to be allocated to other processes, leading to enhanced race performance.

Table 5 outlines the morphological pressures imposed by both locomotion and thermoregulation. While the biomechanical pressures imposed by running economy are constant, the thermal pressures imposed by climate varied by race. As a result, biomechanical and thermal pressures acting upon morphology are aligned in hot conditions, but are in conflict in the cold. As previously discussed, running economy favours long legs and small bodies.

**Table 5.** The energetic pressures imposed on morphology by running economy, Bergmann's Rule and Allen's Rule in hot and cold conditions

	Cold	Hot
Running economy	Longer legs Smaller body	Longer legs Smaller body
Allen's Rule	Shorter legs	Longer legs
Bergmann's Rule	Larger body	Smaller body

### Physical activity driving ecogeographical patterning

The observation that athletes with environmentally adapted morphologies tend to out-perform those lacking such adaptations highlights the significant thermoregulatory challenges of physical activity in extreme environments. Prolonged physical activity heightens the physiological demand for heat loss and heat conservation in hot and cold environments respectively, providing powerful selective forces which could potentially drive the emergence of temperature-adapted morphologies. We propose that it is the interaction between environment and prolonged physical activity that leads to the emergence of environmentally appropriate morphologies, rather than a situation in which adaptation to an environment allows for resultant activity. As described earlier, the interaction between physical activity and thermoregulatory burden may have been more pronounced in hot environments (Ocobock 2016).

The mechanisms underpinning morphological climatic adaptations are unclear. Although the majority of studies have considered natural selection to be the driving force behind climate and ecogeographical patterning (Ashton *et al*. 2000), developmental plasticity may also play an important role (Paterson 1996). Prior to experimental work performed by Serrat *et al*. (2008), the predominant view was that vasomotor changes, which adjust blood nutrient and growth factor supply, were responsible for temperature–growth effects (Trinkaus 1981; Weaver and Ingram 1969). However, vasoconstriction and vasodilation now appear to be influential not because of variation in the delivery of essential growth-related blood constituents, but rather by influencing the temperature within developing cartilage (Serrat *et al*. 2008).

Developmental stress may also play a role. For example, higher birth weights may solve the problem of increased risk of hypothermia early in life in colder settings (Kumar *et al*. 2009; Wells 2002; Wells and Cole 2002). Further support for the concept of developmental plasticity in body proportions stems from work observing heterogeneity linked to sensitivity of different body regions to stress exposure during growth (Payne *et al*. 2018; Pomeroy *et al*. 2012).

The decreasing strength of the relationship between body mass and mean annual temperature during the second half of the twentieth century may provide support for this proposal. While the same negative correlations between body mass and mean annual temperature were observed in 1998 (Katzmarzyk and Leonard 1998) as almost half a century previously (Roberts 1953), the strength of the correlations had declined. The authors explained this as being due to the Westernisation of lifestyle and dietary patterns (Katzmarzyk and Leonard 1998). The transition of a society to become more Westernised is often accompanied by a progressive reduction of habitual physical activity (Rode and Shephard 1994), and this is widely considered to be true of Western societies (Katzmarzyk 2010). It is very possible, therefore, that the recent weakening of the relationship between morphology and environmental temperature is in part due to a reduction in habitual physical activity in thermally challenging environments, reducing the thermodynamic stresses associated with high levels of body mass.

### Limiting factors and future work

The ultra-marathons used in this study were carefully selected to represent as wide a range of environmental conditions as possible (Foster and Collard 2013), and to be consistent in factors such as duration and time spent on the course. However, it was not possible to standardise elevation changes and overall altitude across all race conditions, and different race regulations lead to differences in pack weight carried by competitors. As a result, the biomechanical stresses of running were not entirely consistent across conditions. In the future, an analysis of relationships between morphological variables and performance, including speed, could be analysed in more controlled settings. For example, using treadmills in environmental chambers.

The number of analyses performed here brings potential for Type II errors. However, the morphological traits yielding significant results were consistent across analyses, promoting confidence in the findings.

Athlete questionnaires are required to consider the prevalence of the apparent self-selection based on pre-existing morphologies. Furthermore, it was not possible to control for race time.

Future work should consider the interaction between morphology and performance in female ultra-endurance athletes. Female participation in ultra-endurance athletic events is generally lower than male participation (Knechtle *et al.* 2011). It is hoped that, as female participation in such events increases over the coming years, it will be possible to build a larger database of female competitors.

A greater female sample would also allow for sex-specific analysis of the effects of prolonged endurance activity on physical strength and endurance capacity (as negatively affected during the phenomenon of overtraining) and substrate oxidisation. Analysis of a small number of male athletes completing 86 day and 42 day ski-trek expeditions across Greenland revealed no loss of endurance capacity, a decrease in anaerobic function (Frykman *et al*. 2003) and potential muscle-specific changes in substrate metabolism (Helge *et al*. 2003). Similar studies with female cohorts are necessary.

## Conclusions

Ecogeographical patterning pertaining to Allen's Rule was observed in ultra-marathon runners competing in thermally challenging environments. Heat-adapted morphologies were evident in finishers of hot-condition events relative to non-finishers, and in finishers of hot-condition events relative to finishers of cold-condition events. Furthermore, there appeared to be a degree of niche-picking, by which athletes may have used prior knowledge to avoid exposure to environmental pressures that are detrimental to their performance.

Endurance exercise in hot and cold climates imposes significant thermoregulatory demands, providing powerful selective forces that could drive the emergence of temperature-adapted morphologies. It is proposed that the interaction between environment and prolonged physical activity leads to adaptation and the evolution of environmentally appropriate morphologies. Such morphologies reduce energetic expenditure on thermoregulation, allowing energetic diversion to other processes such as faster running. This is the first study to show a correlation of functional traits with ecogeographical rules.
